# Improved survival among oral cancer patients: findings from a retrospective study at a tertiary care cancer centre in rural Kerala, India

**DOI:** 10.1186/s12957-018-1550-z

**Published:** 2019-01-11

**Authors:** Sajith Babu Thavarool, Geetha Muttath, Sangeetha Nayanar, Karthickeyan Duraisamy, Prasanth Bhat, Kalpita Shringarpure, Priyakanta Nayak, Jaya Prasad Tripathy, Alfonso Thaddeus, Sairu Philip, Satheesan B

**Affiliations:** 1 Department of Surgical Oncology, Malabar Cancer Centre, Thalassery, Kerala 670 103 India; 2 Radiation Oncology, Malabar Cancer Center, Thalassery, Kerala India; 3 Malabar Cancer Centre, Thalassery, Kerala India; 4 Academy of Public Health, Kozhikkode, Kerala India; 5 Department of Health and Family Welfare, Ministry of Health, Bangalore, Karnataka India; 6 Department of Preventive and Social Medicine, Medical College Baroda, Gujarat, India; 7 PATH, Department of International Development, India Country Programme, New Delhi, India; 8 International Union against Tuberculosis and Lung Disease, Paris, France; 9 International Union against Tuberculosis and Lung Disease, The Union South-East Asia Office, New Delhi, India; 10 Department of Community Medicine, Government T.D. D Medical College, Alappuzha, Kerala India

**Keywords:** Oral cancer, Mouth neoplasms, Survival analysis, India

## Abstract

**Background:**

Oral cancer is very common in India. The reported 5-year survival of such patients is around 50% after treatment with surgery and radiotherapy, much lower than most of the developed countries.

**Methods:**

A retrospective study of a prospective database of oral cancer patients undergoing surgery from June 2009 to June 2013 was conducted. Follow-up details were updated from case records and by phone calls. Data were double entered in EpiData Entry version 3.1 and were analysed using EpiData Analysis software 2.1.0.73.

**Results:**

Two-hundred and twenty patients were analysed (136 males); 85% were consuming tobacco, mainly in chewable form. The majority (51.1%) had tongue cancer, of whom 75 patients (34.1%) had T4 tumours. Postoperative radiotherapy was given to 108 patients (49.1%). Forty had recurrence, of which 23 were in early stage. Of these, 19 showed node positivity (*p* < 0.01). Node-negative patients had 79% 5-year survival while node positive had 59% which is comparable to that reported in developed countries. Median disease-free survival duration was 48.2 months.

**Conclusions:**

Node positivity is the single factor affecting recurrence and survival. The overall survival and disease-free survival is better in patients without lymph node involvement and in patients with early stage of cancer as compared to the patients with node involvement and in advanced stages.

## Background

The prevalence of oral cancer is high among the Southeast Asian countries due to the wide use of tobacco products, especially in the chewable form [13]. In India, oral cancer is one of the commonest cancers in both sexes, accounting for 30% of the overall cancer burden, which is likely to increase in the future [18].

Oral cancer patients are treated primarily by surgery in stages 1 and 2 and by surgery with adjuvant therapy in stage 3 and 4 [4]. Patients with positive margin and extra-capsular spread in the nodes are treated with chemoradiation after surgery. Patients with metastasis in nodes, perineural extension and lymphovascular emboli or with advanced tumour stages are treated with adjuvant radiotherapy after surgery [1].

Despite the various treatment modalities available, the overall 5-year survival rate after treatment of oral cancer (all the stages included) is around 50% [12]. Loco-regional recurrence is the most common cause for treatment failure. Recurrence is known to occur in about 35% of patients treated for oral cancer [17]. Recurrent cancer patients have lesser chances of survival [3]. There is little recent literature on survival and recurrence of oral cancer patients in India, most of them being single centre studies. A recent large prospective randomised trial evaluating the effect of elective node dissection versus therapeutic node dissection on survival among oral cancer patients in India showed better overall survival in the elective surgery group (80%) than the therapeutic surgery group (67.5%) at 3 years. However, the study involved only node-negative patients, thus, yielding better outcomes. There exists a gap in literature to identify the factors associated with recurrence in patients treated for oral cancer, in varied Indian settings, where the occurrence of oral cancers is very high.

Kerala is a developed state with the highest literacy rate (around 95%), Human Development Index (HDI) and sex ratio compared to other Indian states. (Census2011) It outperforms other states in terms of health indicators such as low infant and maternal mortality rates. Kerala is one of the top two states reporting highest crude cancer incidence rates, DALYs and deaths closely following Mizoram. One possible reason for this high cancer burden in the state could be the excellent health infrastructure and better community awareness about cancers leading to increased cancer detection.

Malabar Cancer Centre is a tertiary care hospital under the Department of Health and Family Welfare, Government of Kerala, situated in the rural district of Kannur in North Kerala. It provides state-of-the-art oncology care at nominal prices, free services to 20% of patients and concessional rates to around 60% of them. In this unique rural setting in Kerala in a tertiary cancer care centre, we sought to assess the survival among oral cancer patients. The specific objectives were to assess the factors associated with recurrence in oral cancer and to estimate the overall survival and disease-free survival of these patients.

## Methods

This was a retrospective record review conducted among patients with oral cancer who underwent surgery at a tertiary cancer centre in Kerala, from June 2009 to June 2013. All patients were staged according to the American Joint Committee on Cancer (AJCC) staging, seventh edition [5]. Early stage oral cancers were treated by surgery alone and the advanced stage oral cancers by surgery followed by adjuvant therapy. The adjuvant therapy included radiotherapy given with cobalt with three-dimensional planning (for patients enrolled between 2009 and 2012) or with a linear accelerator with VMAT (volumetric modulated arc therapy) with three-dimensional planning (after April 2012).

Patient records were reviewed to extract demographic profile, details of the tumour and surgery which is routinely recorded in the hospital (Appendix). Details of adjuvant therapy were obtained from the radiation chart and case records. Missing data and follow-up data were retrieved and updated from the case records obtained from the medical records department. Follow-up information was obtained from the case record till the last date of follow-up. Any patient not followed up within the last 6 months was contacted over the phone to know their current health status.

Data were double entered into EpiData Entry software, version 3.1, and validated by the principal investigator (SBT) and other co-investigators (GM and SN) to minimise data entry errors. All the discrepancies were noted and corrected by referring back to the original patient records. Data were analysed using EpiData Analysis software version V2.2.2.182 (EpiData Odense, Denmark).

The recurrence of cancer and its pattern was expressed as proportions. The factors associated with recurrence were analysed using chi-square test. The duration of survival was estimated from the date of surgery to the date of last follow-up or date of death. The Kaplan-Meier curve was used to calculate the actuarial probability of overall survival and disease-free survival, and the log-rank test was done to compare the results.

## Results

Of 385 oral cancer patients treated by surgery in the hospital during the study period, eight had histopathologically confirmed non-squamous cell carcinoma. Out of the remaining 377 oral squamous cell carcinoma patients, 152 underwent salvage treatment for oral cancer (had already received radiotherapy outside the hospital without prior surgery). Hence, 220 patients were included in the study, after excluding three who died in immediate post-operative period and two who were lost to follow-up within 2 years.

Majority of the respondents were males (136, 61.8%) and tobacco users (188, 85.5%) with the mean age being 59 years (SD 12.5). The tongue was the most common site of cancer in more than half of the patients (113, 51.4%) followed by buccal mucosa (48, 21.8%) and lower alveolus (34, 15.5%). About 53.6% (*n* = 118) of them were in stages 3 and 4, and the remaining (46.4%, *n* = 102) were in stages 1 and 2 at the time of diagnosis; recurrence was present in 18.2% of the patients (*n* = 40). More than half of the cancers were well differentiated (127, 57.7%) with only 5.0% (*n* = 11) having dysplasia at the margin. Details of the cancer, its spread and management, nodal involvement and other histological parameters are given in Table 1.

**Table 1:** Clinical profile of oral cancer patients who underwent surgery during June 2009–June 2013 at Malabar Cancer Centre, Thalassery, Kerala, India

Variable	Category	*N*	%
Age group (in years)	< 25	01	0.5
	25–44	24	10.9
	45–64	111	50.5
	65 and above	84	38.2
Sex	Male	136	61.8
	Female	84	38.2
Co-morbidity	Absent	143	65.0
	Present	77	35.0
Tobacco use	Absent	32	14.5
	Present	188	85.5
Subsite of primary cancer	Tongue	113	51.4
	Buccal mucosa	48	21.8
	Lower alveolus	34	15.5
	Floor of mouth	09	04.1
	Retromolar trigone	08	03.6
	Upper alveolus	08	03.6
Ipsilateral neck dissection	Selective neck dissection	93	45.1
	Comprehensive neck dissection	113	54.9
Contralateral neck dissection	Selective neck dissection	21	87.5
	Comprehensive neck dissection	03	12.5
T stage of patients	T1	60	27.3
	T2	73	33.2
	T3	12	05.5
	T4	75	34.1
N stage of patients	N0	141	64.1
	N1	30	13.6
	N2a	01	0.5
	N2b	28	12.7
	N2c	07	03.2
	No neck dissection	13	05.9
Extracapsular invasion of node (of 62 positive nodes)	Absent	45	72.6
	Present	17	27.4
Histological differentiation	Well differentiated	127	57.7
	Moderately differentiated	92	41.8
	Poorly differentiated	01	0.5
Margin status	Negative	215	97.7
	Positive	05	2.2
Dysplasia at margin	Absent	209	95.0
	Present	11	05.0
Perineural infiltration	Absent	194	88.2
	Present	26	11.8
Stage of cancer	Stage 1	48	21.8
	Stage 2	54	24.5
	Stage 3	26	11.8
	Stage 4	92	41.8
Postoperative radiotherapy	Not received	112	50.9
	Received	108	49.1
Recurrence status	No	180	81.8
	Yes	40	18.2

One patient with stage 1 and five patients with stage 2 underwent radiation in view of multi-focal perineural invasion. Four patients with stage 2 tongue cancer, with depth more than 1 cm also underwent radiation. Of the 17 patients with extracapsular spread, only 11 were given concurrent chemotherapy and six were not given concurrent chemotherapy in view of poor general condition.

Forty patients had recurrence either loco-regionally or distantly. Seventeen (19.5%) of the advanced stage and 23 (17.3%) of the early stage patients had recurrence. Of the early stage cancers, ten had T1 and 13 had T2 lesions. Of the total 13 patients who had recurrence in the primary site alone, one had pT1 and three had pT2. Among the nine who recurred in ipsilateral neck alone, eight had undergone some form of neck dissection. Two patients who did not undergo neck dissection showed recurrence of the cancer in the neck, of which one had recurrence at the primary site also. Of the 40 patients who recurred, 19 patients had positive nodes after the initial surgery, the association being significant (OR = 2.88, *p* < 0.01); five among them had recurrence in the contra-lateral node. Advanced stage tumours had higher chance of recurrence (OR = 2.33) compared to the early stage tumours. Twenty of the advanced stage patients did not have radiation due to various reasons like deterioration of general condition and unwillingness. Of the five patients having margin positivity, only one patient had recurrence, despite only one patient being given concurrent chemoradiation.

Factors associated with recurrence in oral cancer patients who underwent surgery have been summarised in Table 2. While about one fifth (58, 18%, *p* = 0.8) of tobacco users of any form had recurrence of oral cancer, about a third of those who had ipsilateral node had recurrence (31%, *p* = 0.002). A third of all patients with advanced stage at primary presentation had recurrence (28, 24%, *p* = 0.02).

**Table 2:** Factors associated with recurrence in oral cancer patients who underwent surgery during June 2009–June 2013 at Malabar Cancer Centre, Thalassery, Kerala, India

Variables	Factors		Recurrence status				*p* value
		Total	Present		Absent		
			*N*	%	*N*	%	
Use of Tobacco	Tobacco smoking	90	17	18.9	73	81.1	0.82
	Tobacco chewing	145	28	19.3	117	80.7	0.55
	Use of alcohol	82	13	15.9	69	84.1	0.49
Site of primary	Bucco alveolar	99	15	15.2	84	84.8	0.29
	Tongue/floor of mouth	121	25	20.7	96	79.3	
Neck dissection	Ipsilateral neck dissection	206	38	18.4	168	81.6	0.69
	Selective neck dissection	93	14	15.1	79	84.9	0.25
	Comprehensive neck dissection	113	24	21.2	89	78.8	
	Contralateral neck dissection	24	3	12.5	21	87.5	0.45
Histopathology	Positive margin*	5	1	20	4	80	0.72
	Ipsilateral node positive	61	19	31.1	42	68.9	*0.002*
	Contralateral node positive	6	1	16.7	5	83.3	0.92
	Extra capsular spread	17	6	35.3	11	64.7	0.5
	Perineural invasion	26	7	26.9	19	73.1	0.22
	Advanced stage	118	28	23.7	90	76.3	*0.02*

*Positive margin—reported in final histopathology

The pattern of recurrence in patients with oral cancer who underwent surgery has been summarised in Table 3. Overall, 40 patients had recurrence (18.2%), 31 being loco-regional recurrence and 9 having distant metastasis. Advanced T stage cancers had higher loco-regional and distant metastasis (15% each) compared to early-stage cancers (10.8% and 2.2% respectively). Higher nodal stages (pN2) and extracapsular spread had higher proportions of loco-regional and distant metastasis (37.1% and 35.2% respectively).

**Table 3:** Pattern of recurrence in patients with oral cancer who underwent surgery during June 2009–June 2013 at Malabar Cancer Centre, Thalassery, Kerala, India

Variable		Pattern of recurrence					
		No recurrence		Loco-regional recurrence		Distant metastasis	
		*N*	%	*N*	%	*N*	%
Pathological T stage	Early	81	87.1	10	10.8	2	2.2
	Advanced	14	70	3	15	3	15
Pathological node stage	No neck dissection	11	84.6	2	15.4	0	0
	pN0	123	86.6	14	9.9	5	3.5
	pN1	24	80.0	5	16.7	1	3.3
	pN2	22	62.9	10	28.6	3	8.6
Extracapsular spread	Yes (*n* = 17)	11	64.7	3	17.6	3	17.6
Postoperative radiotherapy	Yes (*n* = 108)	85	78.7	19	17.6	4	3.7
	No (*n* = 113)	95	84.1	13	11.6	5	4.4

**Fig. 1:**
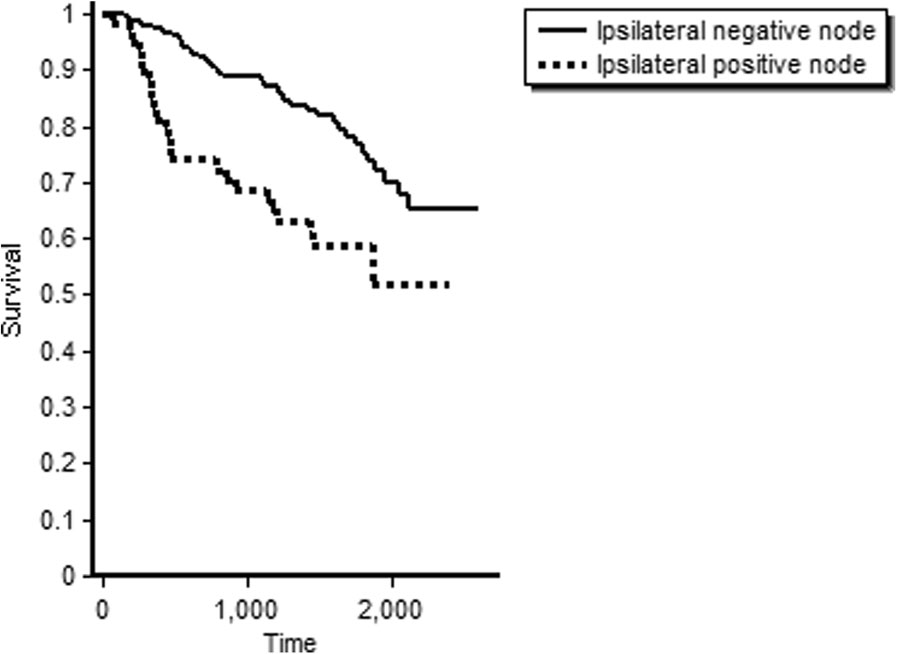
Overall survival of oral cancer patients with positive node and negative node treated at tertiary care cancer centre, India

**Fig. 2:**
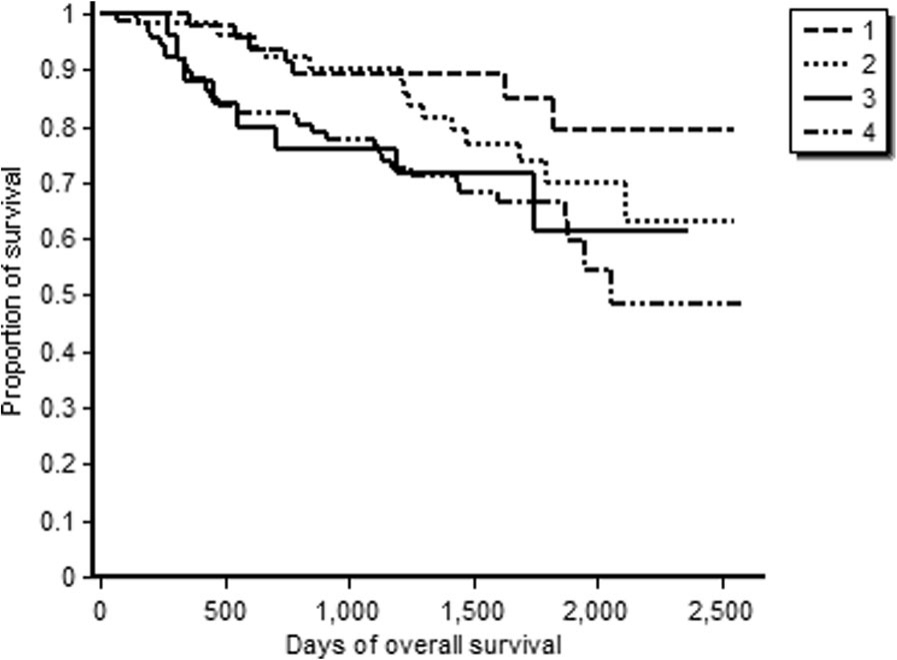
Stage-wise overall survival of oral cancer patients treated at tertiary care cancer centre, India

**Fig. 3:**
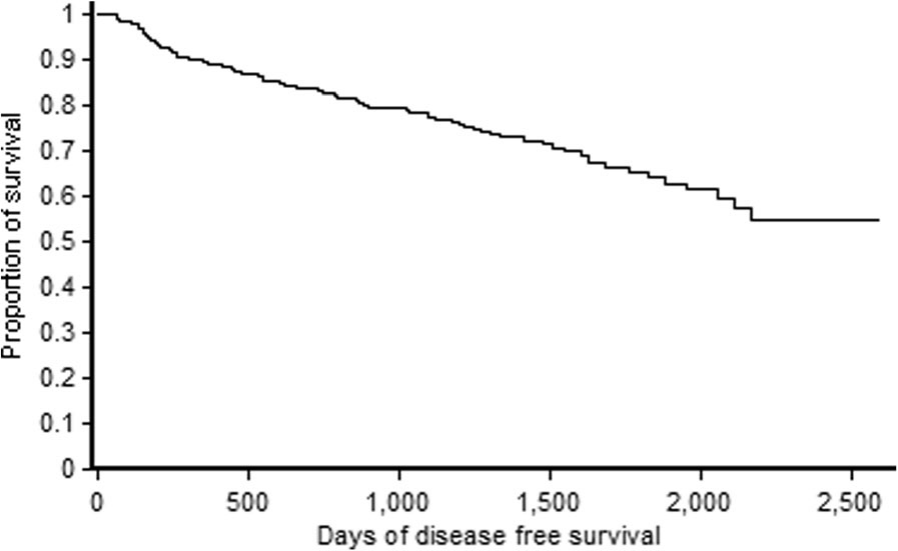
Disease-free survival of oral cancer patients treated at tertiary care cancer centre, India

Kaplan-Meier survival curve for the overall survival of oral cancers in node-positive and node-negative oral cancers is depicted in Fig. 1. The node-positive patients had a lower 5-year survival (59%) whereas the node-negative patients had 5-year survival of 79%. While 58% of those who underwent surgery were alive after 6.8 years, the median duration of survival post-surgery was 50.3 months (IQR 35.6–62.1). The stage-wise overall survival shows that stage 1 tumours had about 86% 5-year survival whereas stage 4 tumours had an overall survival of 62.2% (Fig. 2). Median disease-free survival of the oral cancer patients postoperatively was 48.2 months (IQR 27.3–60.8) (Fig. 3).

## Discussion

In a tertiary cancer care hospital in a rural district of Kerala, this study reported survival among oral cancer patients and risk factors for recurrence in a large cohort. Our study had certain important findings. First, the overall 5-year survival and disease-free survival rates were reasonably good compared to other similar settings elsewhere. Second, the study showed a significant association with the involvement of nodes with cancer recurrence.

The 5-year overall survival and the disease-free survival rates were good in this cohort compared to the average 5-year survival rates observed in published studies from India [19]. The 5-year overall survival from developed countries in all the stages combined ranges from 30 to 70% [2, 11, 14, 16, 20]. Those with early stages (stages 1 and 2) have a better survival rate of up to 80%, whereas patients with advanced stages of cancer have a lower survival rate (30–50%). The type of treatment also has an impact on survival as evidenced in older studies which had more patients treated with radiotherapy. Surgery followed by radiotherapy has become the standard of treatment in oral cancer patients especially in advanced stages [7].

Another reason for better survival and outcome in this cohort could be the fact that nearly half of the patients were in early stage (1 and 2) during diagnosis contrary to other studies where more than 70% of the patients present at an advancing stage. Again, this could be due to better public awareness and affordable and accessible health care system in this unique setting of Kerala.

However, it has been noticed that survival among early-stage cancer patients is 86%, which is marginally less compared to other studies. This might be due to other prognostic factors like depth of invasion (especially among tongue cancer patients) which were not studied in detail in this study and might require future research.

Unique problems exist in the treatment of cancer patients in the majority of the Asian countries which pose a significant barrier to achieving good treatment outcome. Some of these barriers are poverty, illiteracy, advanced stage at presentation, lack of access to health care and poor treatment infrastructure. We believe that one of the possible reasons for better survival in our cohort may be the unique setting that Kerala offers in terms of higher literacy rate, better socio-economic status of the population, improved health infrastructure and access to cancer care, early referral and increased public awareness about health and disease [8]. Nearly half of the patients in this study were in stages 1 and 2 at the time of diagnosis which is a testimony to the abovementioned fact. This is higher compared to other cohorts in India in which 60–80% of patients present with advanced disease. (https://www.hindawi.com/journals/jce/2012/701932/) Nevertheless, survival was also better among patients with stages 3 and 4 which probably reflects the quality of care in the hospital.

A significant factor associated with recurrence was the involvement of nodes, thereby indicating a locally advanced cancer. The patients with advanced stage disease are treated with radiotherapy or chemoradiotherapy after surgery. The involvement of nodes implies that the tumour is aggressive and shows potential for spread. Various factors like advanced stage, deep infiltration, perineural spread and lymphovascular emboli may be the factors determining the nodal spread.

Metastasis to the neck node significantly affected the outcome of patients causing a reduction in survival (68% vs 52% at 6.8 years). All patients with node metastasis have a higher stage of cancer, and hence, advanced stage was also a significant factor predicting the outcome. Though the spread of tumour to the node occurs in predictive pattern from the oral cavity [10], many patients present with an advanced nodal stage. Involvement of node shows the aggressiveness of tumour which is determined by the T stage, depth of tumour, invasiveness, differentiation and hence is an independent factor affecting the overall outcome. Multiple node involvement and lower node involvement significantly affects the survival [9]. The recurrence can occur in ipsilateral neck, contralateral neck or in primary site in advanced stage tumours, and this is independent of the type of neck dissection done [6].

Our study showed that ipsilateral node involvement had significant association with recurrence of the disease. Many of our patients presented with locally advanced cancers due to lack of timely reference and neglect, on part of the patients. Many of these oral cancer patients presented with nodal metastasis in addition to the locally advanced tumour.

The study had several strengths. First, a relatively large cohort of patients with oral squamous cell carcinoma was recruited in this study. Second, there were no missing variables in any of the records and no loss to follow-up as well. Third, the study adhered to the Strengthening Reporting on Observational studies in Epidemiology (STROBE) guidelines and followed sound ethical principles. Fourth, all the records were double entered and cross-validated in EpiData software to minimise data entry errors. Nevertheless, the study had few weaknesses. First, the data regarding the socioeconomic factors were not collected and hence could not be analysed. Second, as the data were from a single tertiary cancer care centre, the results may not be generalizable elsewhere.

The study has important policy implications. First, the results of the study highlight the importance of oral cancer screening for early detection at the primary health care (PHC) setting which may help in improving survival rates. Future studies are recommended to explore the feasibility of cancer screening at the PHC level. Second, those detected at an early stage also require regular follow-up so as to detect and treat complications at the earliest and provide a better quality-of-life to the patients [15]. Third, strategies to improve general public awareness about early detection of oral cancers must be in place.

## Conclusion

The factors affecting the recurrence in oral cancer patients are involvement of nodes along with tumour and advanced stage of the disease. The survival is low in patients with advanced stage of cancer, even after treatment with adjuvant modalities like radiotherapy or chemoradiotherapy which substantiates the role of screening and early diagnosis. The overall survival and disease-free survival is better in patients without nodal involvement and in patients with early stage of cancer as compared to the patients with node involvement and in advanced stages respectively.

## Appendix

### Definitions

Oral cancer: Histopathologically proven squamous cell carcinoma involving any of the subsites like buccal mucosa, alveolus, gingiva, floor of mouth, retromolar trigone, anterior two third of the tongue, or hard palate (excluding lip)

Adjuvant: Treatment which is given after surgery

Performance status: European Cooperative Oncology Group performance score

Primary surgery: Patients who are not treated for cancer previously by surgery, radiation or chemotherapy previously

Status at the time of last follow-up: Whether the patient not having cancer or has local recurrence or has metastasis

Follow-up period: Period in months after completion of treatments

Positive margin: Involvement of tumour at the edge of the resected margin or within 2 mm

Close margin: Involvement of tumour within 2 mm to 5 mm from resected margin

Free margin: No tumour within 5 mm of resected margin

Early cancers: Oral cancers with stage 1 and stage 2 classified after surgery

Advanced cancers: Oral cancers with stage 3 and stage 4 classified after surgery

Recurrence: Occurrence of cancer either at the local site or within 3 cm of the primary site or nodal recurrence in the ipsilateral or contralateral neck after 6 months of completion of treatment and within 3 years after completion of treatment (8)

Local recurrence: Pathologically proven cancer at the site of primary lesion or within 3 cm of the primary lesion

Regional recurrence: Pathologically proven cancer at the ipsilateral or contralateral neck.

Disease-free survival: The duration from the date of surgery to the date of confirmation of “first recurrence”. This does not include the period of disease-free status after the treatment of any recurrence
